# Isolation, Identification, and Antimicrobial Susceptibility Profiles of *Staphylococcus aureus* from Clinical Mastitis in Sebeta Town Dairy Farms

**DOI:** 10.1155/2021/1772658

**Published:** 2021-09-29

**Authors:** Letebrhan Yimesgen W. Grima, Shubisa Abera Leliso, Abebe Olani Bulto, Debebe Ashenafi

**Affiliations:** ^1^National Animal Health Diagnostic and Investigation Center (NAHDIC), Sebeta, Ethiopia; ^2^Addis Ababa University, College of Veterinary Medicine and Agriculture Debebe Ashenafi, Addis Ababa, Ethiopia

## Abstract

A cross-sectional study was carried out in and around Sebeta town dairy farms, Finfinne special zone, Ethiopia, from December 2019 to May 2020 to isolate, identify, and test antimicrobial susceptibility profile of *Staphylococcus aureus* from clinical mastitis. A total of 116 milk samples were purposively collected from 57 lactating cows with clinical mastitis. Isolation and identification of *Staphylococcus aureus* were carried out by using primary and secondary biochemical tests. Besides, Biolog was used for microbial identification systems. To know if the isolates develop resistance to antibiotics, the antimicrobial susceptibility test (ATS) was performed on Mueller-Hinton agar by the disk diffusion method. From a total of 57 lactating cows and 116 teat quarters examined, 21.05% (12/57) and 15.52% (18/116) were positive for *S*. *aureus* from clinical mastitis, respectively. From a total of 116 milk samples collected, 15.52% (18/116) *Staphylococcus aureus* were isolated, and from 11 farms surveyed, about 72.72% (8/11) overall farm prevalence of clinical mastitis due to *S*. *aureus* was recorded. All the 18 *Staphylococcus aureus* isolates were found susceptible to sulphamethoxazole/trimethoprim, erythromycin, gentamicin, ciprofloxacin, and chloramphenicol. However, high level of resistance was observed to common drugs such as penicillin (88.89%, 16/18) and tetracycline (61.11%, 11/18). The observed high level of resistance to penicillin and tetracycline also indicates the need to visit our treatment guidelines for mastitis caused by *Staphylococcus aureus*. Therefore, improved management and early treatment of the cases with drug of choice after the antimicrobial susceptibility test for each specific case can reduce chance of further development of resistance and are imperative to tackle clinical mastitis occurring at Sebeta and other similar farms in Ethiopia.

## 1. Introduction

In Ethiopia, the number of intensive and semi-intensive dairy farms has been increasing from time to time due to urbanization, increased human population, and income growth. However, the management practices of these dairy farms remained traditional. In such dairy farms, mastitis is the predominant disease. Mastitis is the inflammation of the mammary gland mainly due to a bacterial infection, and it is characterized by a variety of local and systemic symptoms. Mastitis could be prevented by implementing proper animal health management systems. However, most of the emerging dairy farms in Ethiopia lack optimum management practices and are predisposed to mastitis [1].

Milk is a major component in the human diet all over the world, but it also serves as a good medium for the growth of many microorganisms, especially pathogenic bacteria. However, health risk to consumers can be associated with milk, due to the presence of zoonotic pathogens and antimicrobial drug residues [2]. The quality of milk may be lowered by a number of factors such as adulteration, contamination during and after milking, and the presence of udder infections [3]. Pathogenic organisms in milk can be derived from the cow itself, the human hand, or the environment [2]. *Staphylococcus aureus* is a versatile pathogen of humans and animals that causes a wide variety of diseases [4].

The bacterium is a colonizer of the skin and mucosae from which it can invade multiple organs. In livestock, *S*. *aureus* is an important cause of mastitis, skin and soft tissue infections (SSTI), and to a lesser extent infection of the locomotory system. Surgical site infections (SSI) in which *S*. *aureus* is isolated have been increasingly reported in small companion animals and horses [5]. *S*. *aureus* is the most prevalent and economically significant pathogen causing inflammatory infections in dairy ruminants [6]. Approximately 30%–40% of all mastitis cases are associated with the bacterium [7].

*Staphylococcus aureus* can get access to milk either by direct excretion from udders with clinical or subclinical *staphylococcal* mastitis or by contamination from the environment during handling of raw milk [8, 9]. When the udder is infected, *S*. *aureus* may be excreted through milk in variable amounts up to 108 CFU/mL [7]. The emergence of antibiotic-resistant bacteria has become a global public health concern affecting human and veterinary medicine [10]. The administration of antibiotics for the feeding of animals for curative purposes or as growth promoters may be a major factor in the selection of antimicrobial-resistant bacteria [11].

The indiscriminate use of antibiotics for the treatment of animal and human diseases as well as preservatives for milk has led to the development of multiple antibiotic resistances, thereby rendering the antibiotic treatment ineffective. *S*. *aureus* has been reported to frequently show multiple antimicrobial resistance patterns [12]. There is no recent study which shows the current prevalence and antimicrobial resistance of *S*. *aureus* originated from cows' milk in Sebeta town, central area of Ethiopia. In addition, previous studies conducted in the area were also limited in number. Thus, this study was aimed to isolate, identify, and determine the antimicrobial susceptibility profiles of *Staphylococcus aureus* from clinical mastitis in the study area and finally recommend farm owners to choose genuine antibiotics which can cure *S*. *aureus* caused clinical mastitis.

## 2. Materials and Methods

### 2.1. Study Area

**Figure 1:**
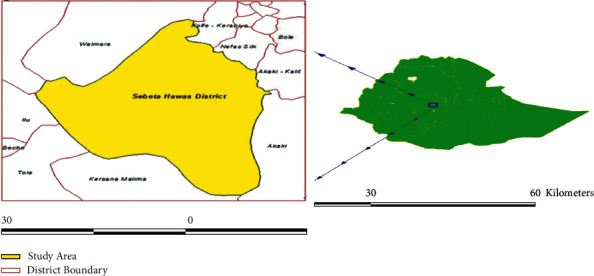
Map of the study area.

The study was conducted in and around Sebeta town, Oromia special zone around Finfinne, Ethiopia. Sebeta is located about 25 km south of Addis Ababa at 8°55′N and 38°37′E and an altitude of 2,356 meters above sea level (Figure 1). The climate is good with an average annual temperature of 17.4°C and average annual rainfall of 1073 mm.

### 2.2. Study Animals

This study was conducted in bovine. Lactating cows of both breeds, crossbreed (Holstein-Friesian-Zebu crosses) and local Zebu breeds, were included during the study period. The study population comprises lactating dairy cows that are managed under semi-intensive and intensive farming systems. A total of 57 lactating dairy cows in and around Sebeta town were sampled to isolate and identify *S*. *aureus* causing clinical mastitis in the area.

### 2.3. Study Design and Sampling

A cross-sectional study was conducted from December 2019 to May 2020 to isolate, identify, and determine antimicrobial susceptibility profiles of *Staphylococcus aureus* in the study area. Eleven (11) dairy farms, 1 big, 4 medium, and 6 small size farms, were randomly selected from Sebeta town. A farm is considered small if the number of animals is <11, medium (11–20), and large (>21). After proper clinical examination of the udder of the cows on the selected farms, cows showing only clinical signs of mastitis were purposively sampled as the aim were to isolate and identify *S*. *aureus* and to know its status on the occurrence of clinical mastitis. As a result, 57 cows were sampled and 116 milk samples from 116 quarters (teats) were collected. However, the remaining 112 quarters (228–116 = 112) were blind and some of them had puss and bloody milk during the sampling time.

### 2.4. Clinical Inspection of the Udder

To identify a mastitis animal, the udders of the cows were examined by visual inspection and palpation for the presence of any lesion, pain, heat, and swelling. In addition, milk from each quarter was withdrawn and checked for any change in colour and consistency [13]. These clinical mastitis cases were diagnosed on the basis of manifestation of visible signs such as inflammation of the udder characterized by heat and swelling with pain upon palpation and/or gross changes in milk, whereas clinical mastitis was diagnosed when misshaped, atrophied, hard, and fibrotic quarters were examined [14]. Accordingly, only cow and udder quarters with clinical cases were selected and from which milk was collected for laboratory testing.

### 2.5. Sample Collection and Handling

Samples were collected aseptically as described in [15]. They were collected before milking. Milk collection process was performed after cleaning the teats, initial stream of milk discarded, and teat tips scrubbed with cotton balls moistened with 75% alcohol. Samples were taken in sterile glass vials and closed with screw caps. The vials were marked with a permanent marker, so that the marking was easy to read when the vials were placed in racks. 10 ml of milk was collected into a horizontally held vial after the first streams of milk were discarded. After collection, the sample was placed in an icebox with +4°C and transported to the National Animal Health Diagnostic and Investigation Center (NAHDIC) for processing and stored at 4°C until inoculation for *S*. *aureus* isolation and identification was started.

### 2.6. Bacterial Identification

**Figure 2:**
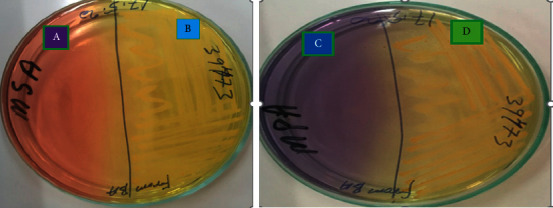
Golden-yellow presumptive colony of *Staphylococcus aureus* on mannitol salt agar (B), fermentation on 1% maltose purple agar base (D), and uninoculated agar parts (A, C).

1 ml of collected milk samples was pre-enriched in 9 ml of brain heart infusion broth incubated at 37°C for 24 hr. A loopful of the incubated culture was streaked onto mannitol salt agar (OXOID) selective and differential media and then incubated aerobically at 37°C for 24 hrs. *S*. *aureus* was identified according to their Gram reaction, morphology, hemolysis, and catalase test. Mannitol fermenting colonies were plated on 1% maltose purple agar base and tested for coagulase (4 h), pigment production (golden yellow) (B in Figure 2), and maltose fermentation (D in Figure 2). Then, OmniLog/Biolog (fully automated coated microplate based bacterial identification system) using GEN III microplate with protocol A method was used to further confirm the species of suspected colonies.

### 2.7. Antimicrobial Susceptibility Test

**Figure 3:**
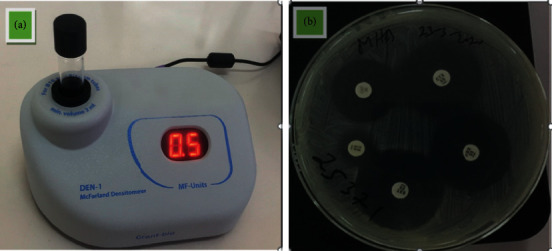
(a) Bacterial cell suspension turbidity measurement using a densitometer, measuring for 0.5 McFarland equivalence. (b) Antimicrobial discs applied on Mueller-Hinton agar by the disk diffusion method.

The antimicrobial susceptibility test (AST) was performed on Mueller-Hinton agar by the disk diffusion method (Figure 3(b)) [16]. Three to five isolated colonies of isolated *S*. *aureus* were transferred to 5 ml of 0.85% saline water. The turbidity was measured using densitometry and adjusted to 0.5 McFarland (Figure 3(a)). After measuring the turbidity, a sterile cotton swab was dipped into the suspension and then Mueller-Hinton agar plate was inoculated by rotating 60°. Antimicrobial discs were applied to the media using a disc dispenser and then incubated for 16–18 hrs.

Measurement of the zone of inhibition was done by using a digital caliper. 10 antimicrobials were selected according to the national AMR surveillance strategic document of Ethiopia [17]. Penicillin G, 10 units; amoxicillin + clavulanic acid (20 + 10), 30 *μ*g; ciprofloxacin, 10 *μ*g; cefoxitin, 30 *μ*g; chloramphenicol, 30 *μ*g; tetracycline, 30 *μ*g; gentamicin, 30 *μ*g; erythromycin, 15 *μ*g; sulphamethoxazole/trimethoprim; cefotaxime, 30 *μ*g (OXOID discs), were used during measuring the zone of inhibition. Standard breakpoints were interpreted based on the Clinical and Laboratory Standards Institute [18], and *S*. *aureus* ATCC 25923 was used as quality control strain in each run.

### 2.8. Data Management and Analysis

Microsoft Excel was used for data management and computation of descriptive statistics. Data were coded and entered on MS Excel spreadsheet. Percentage was calculated by dividing the total number of positive isolates per total number of cows and quarters examined.

## 3. Results

### 3.1. Bacterial Isolation and Identification

From a total of 57 lactating cows and 116 teat quarters examined, 21.05% (12/57) and 15.52% (18/116) were positive for *S*. *aureus* caused clinical mastitis, respectively (Table 1). From a total of 116 milk samples collected, 15.52% (18/116) *Staphylococcus aureus* were isolated from lactating cows having a clinical form of mastitis revealing active cases of mastitis with visible signs of inflammation on the udder and changes in milk quality.

During this study, a total of 11 farms were surveyed and about 8 farms were found positive with *S*. *aureus* (Table 2). Hence, the overall herd prevalence of clinical mastitis due to *S*. *aureus* was recorded to be 72.72%.

### 3.2. Antimicrobial Susceptibility Profiles

Data on the antimicrobial susceptibility of 18 *S*. *aureus* isolates are shown in Table 3. All 18 *S*. *aureus* isolates were susceptible to sulphamethoxazole/trimethoprim, erythromycin, gentamicin, ciprofloxacin, and chloramphenicol. Various rates of resistance, 88.89% (11/18) to penicillin, 61.11% (11/18) to tetracycline, 5.56% (1/18) to cefoxitin, and 5.56% (1/18) to amoxicillin + clavulanic acid, were recorded in this study (Table 3).

Out of the resistant *S*. *aureus* isolates, 2 (11.11%) were found to be multidrug resistant against 3 antibiotic discs primarily to penicillin G, tetracycline, cefoxitin, and amoxicillin + clavulanic acid and 8 (44.44%) isolates were resistant to penicillin G and tetracycline.

## 4. Discussion

The study was conducted on all types (small, middle, and big) of dairy farms in and around Sebeta town to isolate *S*. *aureus* from quarters showing clinical mastitis and determine the prevalence of *S*. *aureus* caused clinical mastitis. The results revealed an overall prevalence of 15.52% at quarter level and 21.05% at cow level *S*. *aureus* caused clinical mastitis.

In the current study, it was noticed that 15.52% of *S*. *aureus* were isolated and identified from quarters with clinical mastitis. Similar results were reported by Esron et al. [4], who reported 15.5% *S*. *aureus* prevalence of clinical mastitis and 13.3% *S*. *aureus* quarter isolates in Addis Ababa dairy farms. The finding of this study was higher than that of previous reports. This higher prevalence might be due to the poor management practiced in the farms. During sample collection time, we observed that they do not follow strict farm biosecurity, proper cleaning of the floor, and washing of animal bodies. Moreover, they do not also exercise the proper milking order which is milking healthy cows (cows with no mastitis), then followed by milking of healthy quarters, and then at last, milking quarters and cows having clinical mastitis.

However, a higher detection rate was reported by other authors [19, 20] who isolated *S*. *aureus* with an incidence of 73.3% and 40.5% at quarter and animal level, respectively, from Addis Ababa dairy farms. High prevalence of *S*. *aureus* is due to its contagious nature and has been adapted to survive in the udder and establish chronic and subclinical infections. From the udder, there is shed of the milk, which serves as a source of infection for healthy cows during the milking process [21].

It is difficult to eliminate the bacteria from the mammary gland due to the very low rate of self-cure and a number of factors affect the rate of cure after treatment, which is in general low [22]. The herd/farm prevalence was also recorded to be high (72.72%) that means from the eleven (11) farms sampled, eight (8) of them were found positive for *S*. *aureus*. This is also due to the poor management practiced in the farms and due to the treatment of clinical mastitis with antibiotics (penicillin G and tetracycline) which have developed resistance to *S*. *aureus*.

Similarly, the current study showed a very high level of resistance at 88.89% and 61.11% for *Staphylococcus aureus* isolates to penicillin G and tetracycline, respectively (Table 3). This high *S*. *aureus* resistance to penicillin G and tetracycline antibiotics might be due the fact that animal health experts in the farm/area do not have other drugs of choice for the treatment of mastitis, instead, they repeatedly use penicillin G and tetracycline during treatment. The finding of this study is similar to [23], who reported 96.7% resistance, and [24], who reported 87.2% resistance. This high level of resistance was due to the isolates producing a penicillinase enzyme (a type of *β*-lactamase) that hydrolyses the beta-lactam ring of penicillin [25].

## 5. Conclusion and Recommendations

The present study showed the occurrence of higher *S*. *aureus* caused clinical mastitis at animal level and very high prevalence at farm/herd levels considering mastitis can be also occurred in subclinical form in farms. Isolates of *S*. *aureus* from clinical mastitis demonstrated the existence of alarming levels of resistance to commonly used antimicrobial agents such as penicillin G and tetracycline suggesting a possible development of resistance to prolonged and indiscriminate usage of those antibiotics. On the other hand, S. *aureus* was also found susceptible to antibiotics such as trimethoprim, erythromycin, gentamicin, ciprofloxacin, and chloramphenicol, suggesting that these antibiotics have not been commonly used as the treatment of choice for mastitis in farms.

On the basis of the above findings, the following recommendations are forwarded:

- Farm management such as farm biosecurity, good farm hygiene, and milking order should be practiced and improved to prevent and control *S*. *aureus* mastitis and to maximize the milk production of the farm
- As a short-term solution, cows detected positive for *S*. *aureus* with clinical mastitis should be treated with erythromycin or gentamicin or ciprofloxacin antibiotics to cure from mastitis and minimize the risk of transmission to other healthy cows
- Implement a systemic application of an *in vitro* antibiotic susceptibility test prior to the use of antibiotics in both treatment and prevention of intramammary infections, which can be considered as a long-term solution to control mastitis
- Regular screening and strip cup examination of the cows and cultural and other bacteriological examination of infected quarters should be conducted so that proper therapy is administered.

## Figures and Tables

**Table 1:** Proportion of *Staphylococcus aureus* isolated from clinical mastitis at cow and quarter levels.

	Samples	Positive	Proportion (%)
Cow level	57	12	21.05
Quarter level	116	18	15.52

**Table 2:** Prevalence of *Staphylococcus aureus* at farm/herd level.

Farm size	Number of farms surveyed	Number of cows sampled	Positive cows	Positive farms	Farm positivity (%)
Big	1	9	3	1	100
Medium	4	34	5	3	75
Small	6	14	4	4	66.6
Total	11	57	12	8	72.72

**Table 3:** Summary result of the antimicrobial sensitivity test (*n* = 18) on isolated *S*. *aureus*.

Antibiotics tested	Susceptible (%)	Intermediate (%)	Resistant (%)
Penicillin G	11.11 (2/18)	0 (0.0)	88.89 (16/18)
Cefotaxime (CXT)	77.78 (14/18)	22.22 (4/18)	0 (0.0)
Cefoxitin (FOX)	94.44 (17/18)	0 (0.0)	5.56 (1/18)
Sulphamethoxazole/trimethoprim (SXT)	100 (18/18)	0 (0.0)	0 (0.0)
Erythromycin (E)	100 (18/18)	0 (0.0)	0 (0.0)
Tetracycline (TE)	38.89 (7/18)	0 (0.0)	61.11 (11/18)
Gentamicin (CN)	100 (18/18)	0 (0.0)	0 (0.0)
Ciprofloxacin (CIP)	100 (18/18)	0 (0.0)	0 (0.0)
Chloramphenicol (C)	100 (18/18)	0 (0.0)	0 (0.0)
Amoxicillin + clavulanic acid (AMC)	94.44 (17/18)	0 (0.0)	5.56 (1/18)

## Data Availability

The data sets used and analyzed during the current study are available from the corresponding author upon reasonable request.
